# Volatiles from a Rare *Acer* spp. Honey Sample from Croatia

**DOI:** 10.3390/molecules15074572

**Published:** 2010-06-24

**Authors:** Igor Jerković, Zvonimir Marijanović, Mladenka Malenica-Staver, Dražen Lušić

**Affiliations:** 1 Faculty of Chemistry and Technology, University of Split, N. Tesle 10/V, 21000 Split, Croatia; 2 Marko Marulić Polytechnic in Knin, P. Krešimira IV 30, 22300 Knin, Croatia; 3 Department of Biotechnology, University of Rijeka, S. Krautzeka bb, 51000 Rijeka, Croatia; 4 Faculty of Medicine, University of Rijeka, Braće Branchetta 20, 51000 Rijeka, Croatia

**Keywords:** *Acer* spp. honey, ultrasonic solvent extraction (USE), headspace solid-phase microextraction (HS-SPME), gas chromatography and mass spectrometry (GC and GC/MS), syringaldehyde

## Abstract

A rare sample of maple (*Acer* spp.) honey from Croatia was analysed. Ultrasonic solvent extraction (USE) using: 1) pentane, 2) diethyl ether, 3) a mixture of pentane and diethyl ether (1:2 v/v) and 4) dichloromethane as solvents was applied. All the extracts were analysed by GC and GC/MS. The most representative extracts were 3) and 4). Syringaldehyde was the most striking compound, being dominant in the extracts 2), 3) and 4) with percentages 34.5%, 33.1% and 35.9%, respectively. In comparison to USE results of other single Croatian tree honey samples (*Robinia pseudoacacia* L. nectar honey, *Salix* spp. nectar and honeydew honeys, *Quercus frainetto* Ten. honeydew as well as *Abies alba* Mill. and *Picea abies* L. honeydew) and literature data the presence of syringaldehyde, previously identified in maple sap and syrup, can be pointed out as a distinct characteristic of the *Acer* spp. honey sample. Headspace solid-phase microextraction (HS-SPME) combined with GC and GC/MS identified benzaldehyde (16.5%), *trans*-linalool oxide (20.5%) and 2-phenylethanol (14.9%) as the major compounds that are common in different honey headspace compositions.

## 1. Introduction

Volatile organic compounds in different honeys often provide a unique aroma profile that constitutes a characteristic fingerprint that may be used for distinguishing honeys as a function of their botanical origin [1,2]. Phenolic compounds, norisoprenoids and terpenoids, as well as aliphatic dicarboxylic acids, have been proposed as strong botanical origin markers of honeys [3,4]. In continuation of the research on honey flavor composition within the scope of chemical characterization (organic analysis) and identification of potential marker compounds, this paper is focused on a Croatian maple (*Acer* spp.) honey sample. *Acer* spp. (such as *Acer campestre *L. *Acer platanoides *L. or *Acer tataricum *L.) are mentioned among the species of Croatian honey-rich plants [5], but in general *Acer* spp. honey is rare in Croatia.

Maple syrup is most widely known as a natural sweetener that is produced by the evaporative concentration of the sap of certain maple species (mainly *Acer saccharum* L. native to the hardwood forests of northeastern North America). The initial maple sap represents a solution in which sucrose is the major component, with minor quantities of reducing carbohydrates and organic acids as well as minerals, nitrogenous and phenolic compounds [6,7,8]. The flavour of maple syrup develops during evaporation. In principle two types of flavour bearing constituents occur: thermal carbohydrate degradation compounds and derivatives of lignin precursor such as coniferyl, dihydroconiferyl, and dihydrosinapyl alcohols. In particular, the lignin derivatives vanillin and syringaldehyde are known to be flavour-bearing constituents of the maple syrup. The concentration of the lignin derivatives does vary considerably (in contrast to the carbohydrates degradation compounds) depending on the provenance and processing [7,8].

No chemical characterization data are currently available for the rare Croatian *Acer* spp. honey, therefore we investigated the volatile compounds of the honey sample in order to identify the most striking ones from the GC and GC/MS results obtained after ultrasonic solvent extraction (USE) with solvents of different polarity and headspace solid-phase microextraction (HS-SPME). The results obtained were compared to other single Croatian tree honey samples as well as literature data.

## 2. Results and Discussion

**Figure 1:**
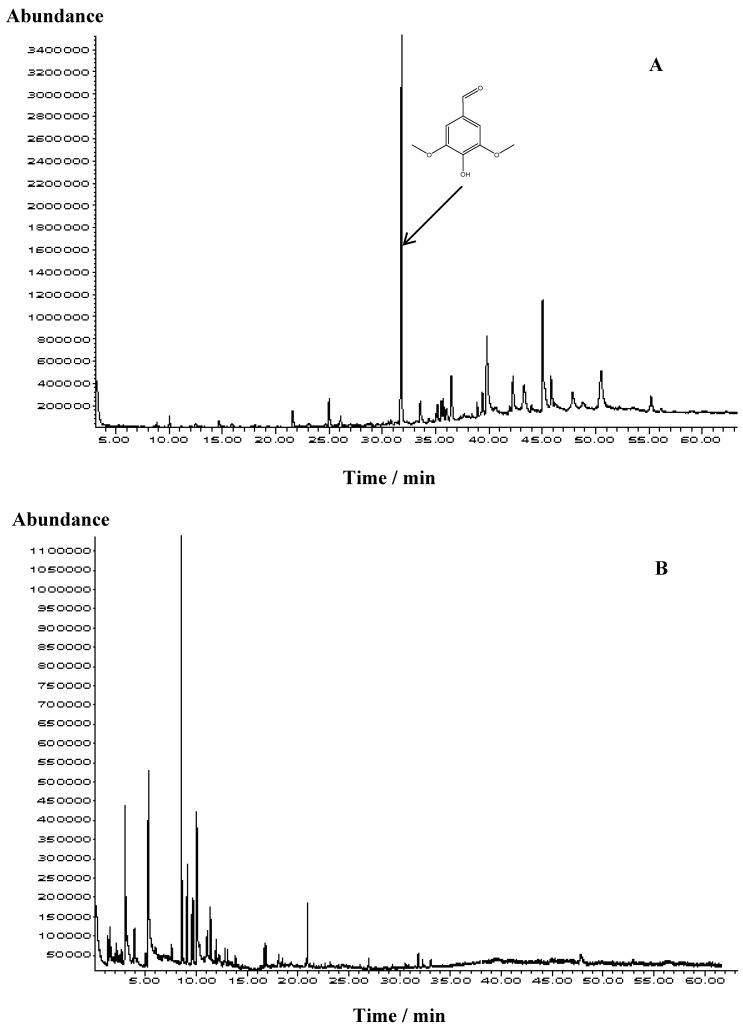
Representative TIC chromatograms of *Acer* spp. honey dichloromethane extract obtained by USE (A) and headspace obtained by HS-SPME (B).

The rare sample of *Acer *spp. honey from Croatia was characterized by melissopalynological analysis: *Acer* spp. pollen was present in an amount of more than 45% in the sediment. The electrical conductivity was measured and amounted 0.32 mS/cm, while water content was determined as 17.5%. Since in previous papers [9,10] we already observed a great variability of the honey headspace and solvent extract volatile composition two methods were used for *Acer* spp. honey volatiles isolation: ultrasonic solvent extraction (USE) and headspace solid-phase microextraction (HS-SPME). Selected TIC chromatograms obtained from GC/MS analyses of representative USE extract and HS-SPME solvent-free extract are presented in Figure 1. Great qualitative and quantitative variability was observed. Identified compounds are listed in Table 1 in accordance to their elution order on an HP-5MS column.

**Table 1:** *Acer* spp. honey volatile organic composition obtained by HS-SPME and USE followed by GC and GC/MS analysis.

No.	Compound	RI	Area percent (%)				
			A	B	C	D	E
1.	Dimethyl sulfide	< 900	1.1	-	-	-	-
2.	2-Methylfuran	< 900	0.8	-	-	-	-
3.	(*E*)-2-Methylbut-2-enal	< 900	1.1	-	-	-	-
4.	2,3-Dihydro-4-methylfuran	< 900	0.6	-	-	-	-
5.	Octane	< 900	0.5	-	-	-	-
6.	2-Furancarboxaldehyde (furfural)	< 900	5.7	-	-	-	-
7.	2-Methylbutanoic acid	< 900	-	-	0.2	-	-
8.	2-Furanmethanol	< 900	-	-	0.2	-	-
9.	4-Methyloctane^*^	< 900	-	0.1	-	-	-
10.	1,4-Dimethylbenzene^**^	< 900	-	0.2	-	-	-
11.	Ethenylbenzene (styrene)	< 900	0.6	-	-	-	-
12.	Heptan-2-ol^*^	< 900	1.4	-	-	-	-
13.	Nonane	900	-	9.3	-	0.1	-
14.	3-Methylpentanoic acid (3-methylvaleric acid)	941	0.6	-	0.3	-	-
15.	Benzaldehyde	965	16.5	0.8	-	-	-
16.	Phenylacetaldehyde	1048	0.8	-	-	-	-
17.	*trans*-Linalool oxide (furan type)	1076	20.5	0.6	-	-	-
18.	4,5-Dimethyl-2-formylfuran	1078	-	-	0.8	-	-
19.	Methyl 2-furoate	1084	-	-	0.6	-	0.3
20.	*cis*-Linalool oxide (furan type)	1091	4.1	-	-	-	-
21.	Linalool	1101	2.0	-	-	-	-
22.	Nonanal	1102	0.7	-	-	-	-
23.	Hotrienol	1106	3.1	-	-	-	-
24.	2-Phenylethanol	1116	14.9	3.4	1.2	0.7	0.9
25.	2,3-Dihydro-3,5-dihydroxy-6-methyl-4H-pyran-4-one	1145	-	-	0.3	-	-
26.	4-Ketoisophorone	1147	0.9	-	-	-	-
27.	Lilac aldehyde (isomer I)^**^	1150	1.4	-	-	-	-
28.	Lilac aldehyde (isomer II)^**^	1158	2.6	-	-	-	-
29.	Benzoic acid	1162	-	-	1.2	0.9	0.2
30.	Pinocarvone	1170	0.6	-	-	-	-
31.	Lilac aldehyde (isomer III)^**^	1173	1.1	-	-	-	-
32.	(*E*)-3,7-Dimethyl-octa-1,5-diene-3,7-diol	1191	-	-	0.3	-	-
33.	Octanoic acid	1193	1.0	-	-	-	-
34.	Dodecane	1200	-	0.3	0.2	-	-
35.	5-Hydroxymethylfurfural	1230	-	-	1.4	0.6	0.7
36.	3-Pyridinecarboxylic acid (nicotinic acid)	1261	-	-	5.6	0.8	-
37.	Phenylacetic acid	1269	-	-	-	1.2	0.3
38.	Nonanoic acid	1273	1.5	-	-	0.2	-
39.	4-Vinyl-2-methoxyphenol	1314	0.9	-	0.4	0.2	0.2
40.	2,4,6-Trimethyphenol^**^	1332	0.4	-	-	-	-
41.	(*E*)-8-Hydroxylinalool	1367	-	-	-	0.2	-
42.	Decanoic acid	1370	0.3	-	-	-	-
43.	*trans*-β-Damascenone	1388	3.1	-	-	-	-
44.	Tetradecane	1400	-	0.9	1.2	-	-
45.	2-Phenylacetamide	1406	-	-	2.4	1.0	2.2
46.	4-Hydroxyphenyl ethanol^*^	1445	-	-	2.7	1.6	0.2
47.	2-Phenylethyl acetate^*^	1492	-	-	3.7	3.2	2.7
48.	Pentadecane	1500	-	-	0.4	-	-
49.	4-Methyl-2,6-bis(1,1-dimethylethyl)phenol	1514	-	0.2	-	0.8	-
50.	N-(2-Phenylethyl)acetamide	1520	-	-	1.4	-	1.0
51.	4-Hydroxybenzoic acid (*p*-salicylic acid)	1522	-	-	1.1	0.8	-
52.	2-Methyldodecan-1-ol^*^	1545	-	0.6	-	0.2	0.3
53.	5-Aminoindanone	1594	-	-	-	-	0.4
54.	Hexadecane	1600	-	0.7	0.7	0.3	-
55.	3-Hydroxy-β-damascone	1617	-	0.4	0.3	0.4	0.3
56.	4-Hydroxy-3,5-dimethoxybenzaldehyde (syringyl aldehyde)	1662	0.2	7.2	34.5	33.1	35.9
57.	(*E*)-3-(4-hydroxy-*m*-methoxyphenyl)-prop-2-enal	1743	-	-	0.6	0.4	0.5
58.	Methyl syringate	1744	-	1.4	1.7	2.2	2.2
59.	α-Hexylcinnamaldehyde^*^	1756	-	-	0.4	0.3	-
60.	3-(4-Hydroxyphenyl)-prop-2-enoic acid (*p*-coumaric acid)	1793	-	-	1.4	0.3	-
61.	Octadecane	1800	-	0.5	0.8	0.4	-
62.	4-Hydroxy-3,5-dimethoxybenzoic acid (syringic acid)	1840	-	-	0.2	-	0.7
63.	Diisobuthyl phtalate	1869	-	2.6	0.8	2.2	1.4
64.	3-(4-Hydroxy-3-methoxyphenyl)-prop-2-enoic acid (ferulic acid)	1874	-	-	1.4	-	-
65.	Hexadecan-1-ol	1882	-	5.0	4.1	4.3	3.0
66.	Hexadecanoic acid	1963	-	2.5	3.7	5.5	6.6
67.	Dibuthyl phtalate	1972	0.7	4.8	0.6	1.6	0.9
68.	(*Z*)-Octadec-9-en-1-ol	2060	-	14.4	3.8	14.9	15.8
69.	Octadecan-1-ol	2084	-	3.3	0.5	4.1	3.6
70.	Heneicosane	2100	-	31.5	3.7	0.2	-
71.	(*Z*)-Octadec-9-enoic acid	2147	-	-	2.1	2.1	2.2
72.	Octadecanoic acid	2181	-	-	-	1.6	0.2
73.	Tetracosane	2400	-	0.3	-	2.5	2.6
		Total identified	89.7	91.0	86.7	88.9	85.3

RI = retention indices on an HP-5MS column; A = solvent-free HS-SPME; B = USE with pentane; C = USE with diethyl ether after pentane extraction; D = USE with mixture of pentane and diethyl ether (1:2 v/v); E = USE with dichloromethane; - = not identified; ^*^ - tentatively identified; ^**^ - correct isomer not identified.

### 2.1. Volatiles Isolated by Ultrasonic Solvent Extraction

Ultrasonic solvent extraction (USE) is a well established procedure for the isolation of honey volatiles at room temperature with the advantage over classical shake-flask extraction of significantly reducing the extraction time. In comparison with other isolation methods this method is particularly appropriate for the isolation of semi-volatile and water-soluble honey compounds [11]. However, optimization of the solvent polarity is necessary, since different chemical compositions can be obtained with more polar and less polar solvents [10,12]. Consequently, four solvents of different polarity were used in order to determine the most appropriate ones for the USE method: 1) pentane, 2) diethyl ether (after pentane extraction), 3) mixture of pentane and diethyl ether (1:2 v/v) and 4) dichloromethane. The obtained pentane extract (Table 1, column B) was composed mainly of non-polar hydrocarbons, as well as higher aliphatic saturated and unsaturated fatty acids and alcohols such as heneicosane (31.5%), (*Z*)-octadec-9-en-1-ol (14.4%), nonane (9.3%) or hexadecan-1-ol (5.0%). All these compounds are, in general, found in different honeys [13]. Syringaldehyde occurred with lowest percentage (7.2%) in comparison with the other extracts. After non-polar compounds were removed by pentane extraction, diethyl ether was applied for further USE of the same sample batch, allowing isolation of more polar honey constituents. In the diethyl ether extract the major identified compound was syringaldehyde (34.5%), followed by minor percentages of the structurally related compounds methyl syringate (1.7%) and syringic acid (0.2%). Great variability of the pentane and diethyl ether extracts composition is noted from Table 1 (columns B and C). The mixture of pentane and diethyl ether (1:2 v/v) isolated syringaldehyde (33.1%) and methyl syringate (2.2%), followed by higher fatty alcohols (*Z*)-octadec-9-en-1-ol (14.9%), hexadecan-1-ol (4.3%) and octadecan-1-ol (4.1%) (Table 1, column D). A similar chemical composition was obtained by dichloromethane extraction (Table 1, column E): syringaldehyde (35.9%), (*Z*)-octadec-9-en-1-ol (15.8%) and hexadecan-1-ol (3.0%). From the obtained results it can be noted that the mixture of pentane and diethyl ether and dichloromethane are the most suitable solvents for *Acer* spp. USE, while pentane cannot be used since a significantly lower percentage of syringaldehyde was identified in comparison with the other solvents.

Syringaldehyde is the most striking compound of *Acer* spp. honey volatiles being dominant in all the extracts except for the non-polar pentane extract. It was not found in other single samples of Croatian tree honeys (Table 2) or in other Croatian honeys [9,10,11,12,13], although it was previously identified in maple sap and syrup [8], suggesting its good potential as an *Acer* spp. honey marker compound. 

**Table 2:** Comparison of major volatile constituents of *Acer* spp. honey sample and other representative Croatian tree honey samples obtained by USE with the solvent pentane-diethyl ether (1:2 v/v) followed by GC and GC/MS analysis.

No.	Compound	RI	Area percent (%)					
			*Acer *spp.	*Pseudoacacia robinia* L.	*Salix *spp.	*Salix* spp. honeydew^a^	*Quercus frainetto* Ten.honeydew^b^	*Abies alba* Mill. and *Picea abies* L. honeydew
1.	1-(2-Furyl)-2-hydroxy-ethanone	914	-	-	-	-	5.1	
2.	Phenylacetic acid	1273	-	-	10.2	0.2	16.4	3.8
3.	4-Vinyl-2-methoxyphenol	1314	0.2	3.3	-	-	-	-
4.	4-Hydroxy-2-phenyl-ethanol	1429	-	3.1	0.6	1.2	0.6	-
5.	2-Phenylethyl acetate^*^	1492	3.2	-	-	-	-	-
6.	4-Hydroxyphenylacetic acid	1563	-	-	-	12.7	-	8.9
7.	Syringyl aldehyde	1662	33.1	-	-	-	-	-
8.	Methyl syringate	1744	2.2	-	-	-	4.6	-
9.	4-Hydroxycinnamic acid	1817	-	-	-	0.8	6.6	1.5
10.	Hexadecan-1-ol	1882	4.3	8.9	2.1	2.8	2.0	2.1
11.	Hexadecanoic acid	1963	5.5	12.1	1.4	5.4	1.7	4.6
12.	8-Hydroxy-4,7-dimethylcoumarin	2203	-	-	7.4	-	-	-

RI = retention indices on an HP-5MS column; - = not identified; ^*^ - tentatively identified;

^a^ - reference [12]; ^b^ - reference [16].

It has been suggested that the source of vanillin and syringaldehyde in maple syrup is lignin, and it is well known that syringaldehyde is formed by the degradation of lignin as an oxidation product of sinapaldehyde [14]. Later, Potter and Fagerson [15] reported the identification of phenolic lignin monomers and related flavor compounds in dichloromethane extracts of maple syrup. Different kinds of phenolic compounds were found from maple sap, concentrates of the sap and the syrup [7]: vanillic acid, syringic acid, homovanillic acid, coniferyl alcohol, vanillin, *p*-coumaric acid, syringaldehyde, sinapic acid, ferulic acid and coniferylaldehyde. Besides syringaldehyde, several of these compounds such as syringic acid (0.0-0.2%), *p*-coumaric acid (0.0-1.4%) or ferulic acid (0.0-1.4%) were also identified as minor components in *Acer* spp. honey.

### 2.2. Volatiles Isolated by Headspace Solid-Phase Microextraction

Headspace solid-phase microextraction (HS-SPME) combined with GC and GC/MS enabled identification of the most volatile organic headspace compounds of *Acer *spp. honey (Table 1, column A). The most abundant compounds in the headspace were the ubiquitous honey benzene derivatives benzaldehyde (16.5%), 2-phenylethanol (14.9%) and phenylacetaldehyde (0.8%). The headspace percentages of terpenes were high: *trans*-linalool oxide (20.5%), *cis*-linalool oxide (4.1%), linalool (2.0%) or hotrienol (3.1%), followed by three lilac aldehyde isomers (1.1-2.6%). Two norisoprenoids were also found [4-ketoisophorone (0.9%) and *trans*-β-damascenone (3.1%)], as well as several lower aliphatic acids [octanoic acid (1.0%), nonanoic acid (1.5%) and decanoic acid (0.3%)]. From the obtained results no specific *Acer* spp. headspace compound can be pointed out as a potential marker, since all these compounds have been reported as common constituents of various honeys [1,2,3]. 

## 3. Experimental

### 3.1. Honey Sample

The sample of *Acer* spp. honey was obtained from an area of wild growing *Acer *spp. and no mechanical treatment or heat was used. The honey samples of *Pseudoacacia robinia* L., *Salix* spp., *Abies alba* Mill. and *Picea abies* L. honeydew were obtained from professional beekeepers. All the samples were stored in hermetically closed glass bottles at 4 ºC until the volatiles isolation. Melissopalynological analysis was performed by the methods recommended by the International Commission for Bee Botany [17]. Microscopical examination for morphometry of pollen grains was carried out on a Hund h 500 (Wetzlar, Germany) light microscope attached to a digital camera (Moticm 1000) and coupled to an image analysis system (Motic Images Plus software). Water content was determined by refractometry using a standard model Abeé refractometer at 20 ºC. Water content (%) was obtained from the Chataway table [18]. Electrical conductivity was measured in a solution of 20 g honey sample in low conductivity water at 20 ºC using a conductometer (Hanna HI 8733). 

### 3.2. Ultrasonic Solvent Extraction (USE)

Ultrasound-assisted solvent extraction (USE) was performed in an ultrasound cleaning bath (Elmasonic Typ S 30 H, Germany) in the indirect sonication mode (sweep mode), at the frequency of 37 kHz at 25 ± 3 ºC. Forty grams of the sample was dissolved in distilled water (22 mL) in a 100-mL flask. Magnesium sulfate (1.5 g) was added and each batch of *Acer* spp. honey sample was extensively vortexed. Different solvents were used: 1) pentane 2) diethyl ether (after pentane extraction and removing the pentane layer on the sample batch), 3) mixture of pentane and diethyl ether (1:2 v/v) and 4) dichloromethane. The solvents were used separately for the extraction of the honey sample batch. The extraction of other honey samples was performed with a mixture of the solvents pentane and diethyl ether (1:2 v/v). Sonication was maintained for 30 min. After sonication, the organic layer was separated by centrifugation and filtered over anhydrous MgSO4. The aqueous layer was returned to the flask and another batch of the same extraction solvent (20 mL) was added and the mixture extracted by ultrasound for 30 min. The organic layer was separated in the same way as the previous one and filtered over anhydrous MgSO4, and the aqueous layer was sonicated a third time for 30 min with another batch (20 mL) of the extraction solvent. The combined organic extracts were concentrated to 0.2 mL by distillation with a Vigreaux column, and 1 μL was used for GC and GC/MS analyses.

### 3.3. Headspace Solid-Phase Microextraction (HS-SPME)

The isolation of headspace volatiles was performed using a manual SPME fiber with a layer of divinylbenzene/carboxen/polydimethylsiloxane (DVB/CAR/PDMS) obtained from Supelco Co (Bellefonte, PA, USA). The fiber was conditioned prior to use according to the manufacturer’s instructions. For HS-SPME extraction, honey/saturated water solution (5 mL, 1:1 v/v; saturated with NaCl) was placed in a 15 mL glass vial and hermetically sealed with a PTFE/silicone septum. The vial was maintained in a water bath at 60 ºC during equilibration (15 min) and extraction (45 min) under constant stirring velocity (1,000 rpm) with a magnetic stirrer. After sampling, the SPME fiber was withdrawn into the needle, removed from the vial, and inserted into the injector (250 ºC) of the GC and GC/MS for 6 min where the collected volatiles were thermally desorbed directly to the GC column.

### 3.4. Gas Chromatography and Mass Spectrometry (GC, GC/MS)

Gas chromatography analyses were performed on an Agilent Technologies (Palo Alto, CA, USA) gas chromatograph model 7890A equipped with a flame ionization detector and a quadropole mass spectrometer model 5975C. A capillary column HP-5MS [(5%-phenyl)-methylpolysiloxane Agilent J & W GC column, 30 m, 0.25 mm i.d., coating thickness 0.25 μm] was used. Chromatographic conditions were as follows: helium was carrier gas at 1 mL·min^−^^1^, injector temperature was 250 ºC, and FID detector temperature was 300 ºC. The temperature used included the following settings: 70 ºC isothermal for 2 min, and then increased to 200 ºC at a rate of 3 ºC·min^−^^1^ and held isothermal for 18 min. The injected volume was 1 μL and the split ratio was 1:50. MS conditions were: ionization voltage 70 eV; ion source temperature 230 ºC; mass scan range: 30–300 mass units. The analyses were carried out in duplicate.

### 3.5. Data Analysis and Data Evaluation

The individual peaks were identified by comparison of their retention indices (relative to C_9_-C_25_ *n-*alkanes for HP-5MS) to those of available authentic samples and literature values [19], as well as by comparing their fragmentation patterns with those found in the Wiley 275 MS library (Wiley, New York, NY, USA) and NIST02 (NIST, Gaithersburg, MD, USA) mass spectral database. The percentage composition of the samples was computed from the GC peak areas using the normalization method (without correction factors). The component percentages were calculated as mean values from duplicate GC and GC-MS analyses.

## 4. Conclusions

Research on volatiles of a rare Croatian *Acer* spp. honey sample indicating syringaldehyde as the major component has suggested further detailed analysis of more *Acer* spp. samples from different origins after solvent extraction with polar solvents and examination of semi-volatiles. Typical honey headspace volatiles (benzene derivatives, terpenes, several norisoprenoids and others) were also identified. In comparison to the results obtained from other Croatian tree honey samples and literature data, it appears that syringaldehyde may be a distinctive compound of *Acer* spp. honey, although analyses of more samples of this rare honey are needed for confirmation of this fact.
